# Seeing and Hearing a Word: Combining Eye and Ear Is More Efficient than Combining the Parts of a Word

**DOI:** 10.1371/journal.pone.0064803

**Published:** 2013-05-29

**Authors:** Matthieu Dubois, David Poeppel, Denis G. Pelli

**Affiliations:** 1 Psychology and Neural Science, New York University, New York, New York, United States of America; 2 Cognition and Neuroscience Research Center, Université Libre de Bruxelles, Brussels, Belgium; 3 Fonds National de la Recherche Scientifique (FRS-FNRS), Brussels, Belgium; Max Planck Institute for Human Cognitive and Brain Sciences, Germany

## Abstract

To understand why human sensitivity for complex objects is so low, we study how word identification combines eye and ear or parts of a word (features, letters, syllables). Our observers identify printed and spoken words presented concurrently or separately. When researchers measure threshold (energy of the faintest visible or audible signal) they may report either sensitivity (one over the human threshold) or *efficiency* (ratio of the best possible threshold to the human threshold). When the best possible algorithm identifies an object (like a word) in noise, its threshold is independent of how many parts the object has. But, with human observers, efficiency depends on the task. In some tasks, human observers combine parts efficiently, needing hardly more energy to identify an object with more parts. In other tasks, they combine inefficiently, needing energy nearly proportional to the number of parts, over a 60∶1 range. Whether presented to eye or ear, efficiency for detecting a short sinusoid (tone or grating) with few features is a substantial 20%, while efficiency for identifying a word with many features is merely 1%. Why? We show that the low human sensitivity for words is a cost of combining their many parts. We report a dichotomy between inefficient combining of adjacent features and efficient combining across senses. Joining our results with a survey of the cue-combination literature reveals that cues combine efficiently only if they are perceived as aspects of the same object. Observers give different names to adjacent letters in a word, and combine them inefficiently. Observers give the same name to a word’s image and sound, and combine them efficiently. The brain’s machinery optimally combines only cues that are perceived as originating from the same object. Presumably such cues each find their own way through the brain to arrive at the same object representation.

## Introduction

Object recognition is the most important perceptual task. Since Fechner, sensitivity has been measured to characterize how people recognize objects [1], [2]. There is growing conviction that observers recognize an object by detecting and combining its features [3], [4], [5], [6]. But how do we combine? Multi-sensory perception is essential to most everyday activities [7], [8], [9], [10], [11], [12], spurring work ranging from physiology [13], [14], [15] to therapeutic educational interventions [16], [17], [18], but, once again, now across senses, how we combine remains mysterious. Cue combination has been studied in many tasks at various levels [19]. Here we focus on word identification, at the levels of features, letters, syllables, and words, within and across senses. We report a dichotomy in word identification between inefficient combining of adjacent features and efficient combining across senses. For example, observers combine adjacent letters in a word inefficiently, but combine the sound and image of a word efficiently. Joining our new results with a survey of the cue-combination literature reveals that cues combine efficiently if and only if they are perceived as aspects of the same object. By an “object,” we mean something recognizable, often with a name, e.g. a border, a bar, a plane, a grating, a letter, a word, a dancer.

Physicists define the efficiency of a motor as the fraction of the energy provided to the motor that it returns as useful work. Psychophysicists define the *efficiency* of a human observer as the fraction of the signal energy used by the observer that the optimal algorithm (the ideal observer) would need to perform the recognition task [20], [21], [22], [23]. Efficiency tells us how well the observer recognizes signals in noise. If we were more efficient, we would be more sensitive. We could hear our friends in a noisy pub, and we could read outdoors late into the evening.

Audio and visual efficiencies have previously been measured, but, oddly, no one has ever compared them. We show that eye and ear have the same efficiency, whether detecting a short sinusoid or identifying a word. The equal efficiencies of eye and ear, noted here for the first time, hint that object recognition may involve similar computations in the auditory and visual systems. Is there something general about word recognition that transcends eye and ear? Are the computations similar? “Relative efficiency” [23], introduced below, helps us move forward by allowing a piecemeal approach, breaking up the big question into several smaller questions. It allows us to express the overall efficiency as a product of several factors that we can figure out one at a time.

For any given number of possible signals, if signal energy is fixed, the optimal algorithm (ideal observer) recognizes a long signal just as easily as a short one. This was proven in the 1950’s for detection [21], [22], and more recently for identification [24], [25]. In a noisy room, a brief shout and a long whisper with equal energy are equally detectable (by the optimal algorithm), but, as we will see below, people are much better at hearing the briefer one. Unlike the ideal, human observers require more energy to recognize longer words, i.e. with more parts, because the human observer combines inefficiently.

*Combining* is the integration of information from several parts to get information about the whole. This also has been called “summation” or “integration.” The closely related terms “binding,” “segmentation,” and “grouping” emphasize selecting which parts are to be combined. Combining occurs at every level of perceptual processing. Our new approach applies Geisler’s [23] general notion of “relative efficiency” (a ratio of efficiencies) to our specific problem of assessing combining. We do this by measuring how much more energy the observer needs to recognize a signal with more parts (e.g. short vs. long words, with more printed letters or spoken syllables, or 1 vs. 2 sense modalities). We define *summation efficiency* as the ratio of the efficiency for a signal with more parts to that for a signal with fewer parts. Recall that efficiency is the ratio of ideal to human threshold energies. If the task is appropriately designed, so that the ideal energy threshold is independent of the number of parts, then the ideal threshold cancels out in the ratio of efficiencies, leaving a ratio of two human thresholds.

We measure the threshold sound energy in hearing and contrast energy in vision. In all our experiments, the noise is independent among all the parts. If the observer combines perfectly, and the task is appropriately designed, then the observer will require the same energy, independent of the number of parts. If the observer combines inefficiently, then the observer will require more energy for more parts [24]. We show that our new measure, summation efficiency, is a useful assay of the cost of combining. While past approaches have not provided much grip on this key but slippery phenomenon, we note that our summation efficiency is very closely related to, but easier to understand than Nandy and Tjan’s “index of integration” [26].

### Summation Efficiency

*Efficiency* is the ratio of ideal and human energy thresholds, *η* = *E*_ideal_/*E*. Let *η*_1_ be the efficiency for identifying one feature. We do not know its value. On general grounds, one might suppose that it is less than 100% and greater than or equal to the 20% found for detecting a brief sinusoid (both in hearing [27] and in vision [28]), which presumably has only a few if not just one feature (see ‘Efficiency to detect a short sinusoid’ in Materials and Methods). Let *η* be the efficiency for identifying an *n*-feature word. The *summation efficiency* is 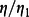.

Human and ideal observers differ in how well they combine parts, but are alike in many other ways. The relation of human to ideal sensitivity is efficiency. When human and ideal are similarly affected, efficiency is unchanged. In fact, efficiency is independent of overall contrast, eccentricity, and, to some extent, task (e.g. number of response alternatives) [25]. Detection is fundamentally easier than identification because there are fewer response alternatives, but this is factored out in computing efficiency, so efficiency for identification of one of many possible can equal the efficiency for detection of one.

#### Summation index

A traditional formula describing psychophysical summation of multiple components is
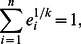
(1)where *n* is the number of components, *k* is the *summation index*, *e_i_* is the energy of the *i*-th component, normalized by the threshold 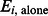 for that component alone, 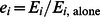, and the composite stimulus is at its threshold (*e*_1_, …, *e_n_*). If all the components are presented at the same multiple of threshold, 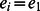 for all *i*, then Eq. 1 reduces to 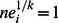 so



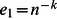
(2)When the limiting noise is independent and identically distributed among the components, then summation ranges from none (*k = *0) to full (*k = *1). Note that Eq. 1 and the index *k* apply to energy, not amplitude. This ideal, with full summation (*k = *1), is the “integrating model” of Green and Swets [22]. The ideal observer summates fully (*k = *1) and humans summate weakly or strongly, depending on the task (Table 1). Suppose the task is designed so that the ideal total energy threshold *E*_ideal_ is independent of the number of components. This is easy to do. Then the summation efficiency is.
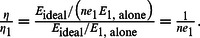
(3)


**Table 1:** A survey of summation.

Publication	Senses		*k* for ‘same’ object	*k* for ‘different’ objects	‘Same’ object	‘Different’ objects	Report	Cues
**This paper**	2	Vision & hearing	0.76		A word, seen and heard		Word	Spoken and printed word
**Arrighi et al. [53, Exp. 2]**	2	Vision & hearing	0.68	0.48	Point-light tap dancer, seen and heard	Two different point-light tap-dance movies, one seen and one heard	Dancing presence	Dot display and sound of tap dancing plus random dots and taps
**Meyer et al.** [**54**]	2	Vision & hearing	0.7	0.25	A moving flash/click, seen and heard	A moving flash and a moving click, displaced	Motion	31 lamps and loudspeakers along the horizontal meridian presenting flashes and clicks that move
**Alais and Burr ** [**8**]	2	Vision & hearing	1		A blob-click, seen and heard: “a ball thudding onto the screen”		Location	A brief bright visual blob and a sound click
**Gori et al. [55,** **Fig. 4** **]**	2	Vision & tactile	1		Moving grating, seen and felt		Velocity	Visual and tactile motion of metal gratings
**Gepshtein et al.** [**51**] **; Gepshtein and Banks** [**52**]	2	Vision & haptic	0.74	0.17	Like a thick dusty sheet of glass, seen and felt	Two glass sheets, displaced, one seen and one touched	Thickness (distance between the two parallel surfaces)	Seeing random dot visual stereogram of front and back surfaces and squeezing the two surfaces between thumb and fingertip
**Ernst and Banks ** [**7**]	2	Vision & haptic	1		A bar, seen and felt		Thickness	A raised bar in a visual stereogram and a bar squeezed by finger and thumb
**Hirsh et al.** [**48**]	1	Hearing		0.46		The syllables of a word	Word	Spoken syllables, successive
**Rubenstein et al.** [**49**]	1	Hearing		0.54		The syllables of a word	Word	Spoken syllables, successive
**Green et al.** [**56**]	1	Hearing		0.53		16 different simultaneous tones	Tone presence	Brief tones of 16 different frequencies
**Pelli et al.** [**24**]	1	Vision		0.1		The letters of a word	Word	Printed letters, adjacent
**Pelli et al.** [**25**]	1	Vision		0.1		The “features” of a letter	Letter	One of 26 letters of a given font or alphabet. The number of “features” is assumed to be proportional to letter complexity.
**Näsänen et al.** [**57**]	1	Vision		0.48		The “features” of a letter	Letter presence	A band-pass filtered letter. The number of “features” is assumed to be proportional to letter complexity.
**Nandy and Tjan ** [**26**]	1	Vision	1		A letter, filtered to remove all but a band of high or low spatial frequencies		Letter	High- and low-frequency bands of a letter
**Nandy and Tjan [26, Appendix D]**	1	Vision		0		High- and low-frequency gratings	Grating presence	High- and low-frequency gratings
**Graham et al.** [**33**]	1	Vision		0.57		High- and low- frequency gratings	Grating presence	High- and low-frequency gratings
**Robson and Graham ** [**29**]	1	Vision		0.57		Grating patches, adjacent	Grating presence	Grating patches, adjacent
**Watson ** [**58**]	1	Vision		0.43		The many brief gratings that make up a prolonged grating	Grating presence	A grating of various durations
**Rovamo et al.** [**59**]	1	Vision		0.30		Successive cycles over time	Flicker presence	A circular uniform field flickering at various temporal frequencies
**Watson et al.** [**60**]	1	Vision	0.95	0.58	A “stationary” grating, actually two gratings moving very slowly in opposite directions	Two gratings moving quickly in opposite directions	Grating presence	Two gratings moving in opposite directions
**Knill and Saunders** [**61**] **; Hillis et al.** [**62**]	1	Vision	1		A slanted plane, with appropriate gradients in texture and binocular disparity		Surface slant discrimination	A plane whose slant is cued by the perspective gradient in texture, or by the gradient in binocular disparity, or both
**Oruç et al.** [**63**]	1	Vision	1		A slanted plane, with appropriate linear perspective, or perspective-caused gradient in texture, or both		Surface slant	Linear perspective and texture gradient
**Rivest and Cavanagh ** [**64**]	1	Vision	0.89		The border between two areas		Location	Boundary contour between two areas differing in luminance, color, and/or texture.

Taken from 23 papers, these are *summation* experiments: the observer’s thresholds for a compound stimulus are compared with the thresholds for the components of the compound. [31, Sec. 1.11.2]. The index of summation, *k*, ranges from none (*k = *0) to optimal (*k = *1). This table shows that, whether using one or several senses, whether the task is high- or low-level, the summation is strong (*k* near 1) only if the two components are both perceived as aspects of the “same” thing. When cues are “different” things, summation is weak 0≤ *k* ≤0.58 with a mean 0.37; when cues are “same”, summation is strong 0.68≤ *k* ≤1 with a mean 0.89. This table is based on 23 papers, including all the perceptual summation efficiencies for adults that we found (or could calculate from published results) in a quick survey of the literature. It includes only summation of cues that are consistent and informative. It omits cue conflict studies, in which the cues provide conflicting information about the quality to be reported. It also omits the facilitation paradigm, in which one of the cues provides no information. At an *n*-component threshold that equates the energy of the components, if the summation index is *k* then the summation efficiency is_251658240_. Conservation of energy, i.e. the optimal algorithm, has *k* = 1. Independence of successes, i.e. probability summation, has *k* ≈ 0.57. In most cases the papers do not report whether their observers perceived the two components as the “same” object, so we have guessed, based on our experience and a close reading of what the papers do say. Another two papers turned up by our search could not be included in the Table because we were unable to classify their stimuli, with any confidence, as “same” or “different” [45], [65]. Obviously our guesses must yield to better assessments. Table S1 explains how *k* was estimated for each paper. Note that the predicted value for the summation index of roughly 0.6 for probability summation is for detection, whereas most of the experiments in Table 1 are identification.

Using Eq. 2 to replace *e*_1_, we get
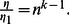
(4)


We can solve this for *k*, the summation index,
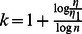
(5)


#### Factoring efficiency

The efficiencies are nonzero, so we can write
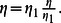
(6)


This is the product of identification efficiency for one feature, *η*_1_, and summation efficiency for *n* features, *η*/*η*_1_. We noted that a summation index *k* implies a summation efficiency *η*/*η*_1_ = *n^k^* ^−1^. Thus
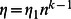
(7)where 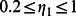. Eq. 7 is a key prediction. Previous observations that this is a power law did not mention that the proportionality constant is the efficiency for one feature [24], [25]. Eq. 7 says that the highest efficiency *η*_1_ is attained when detecting just one feature (*n = *1). We will see in Results and Discussion that summation is strong (*k* near 1) only for combining components that are perceived as coming from the same object. Efficiency is low when many features are summed weakly (*n* >>1 and *k* <<1).

Our derivation began with an empirical law (Eq. 1). However, that law, for detection or identification, can be derived by supposing that objects consist of features that are detected independently; that detecting the object merely requires detecting a feature; and that identifying the object requires detecting several (perhaps 7) features [25].

### Summation Models

Components can be closely related (e.g. adjacent pixels) or disparate (e.g. auditory and visual). Adjacent pixels are highly correlated in most environments, as are successive sounds, whereas audio is correlated to visual only in particular situations. Given multiple sources of information with independent noise, how do we combine them to arrive at a single decision? From a computational perspective, there is a range of possibilities, but two cases stand out.

**Figure 1:**
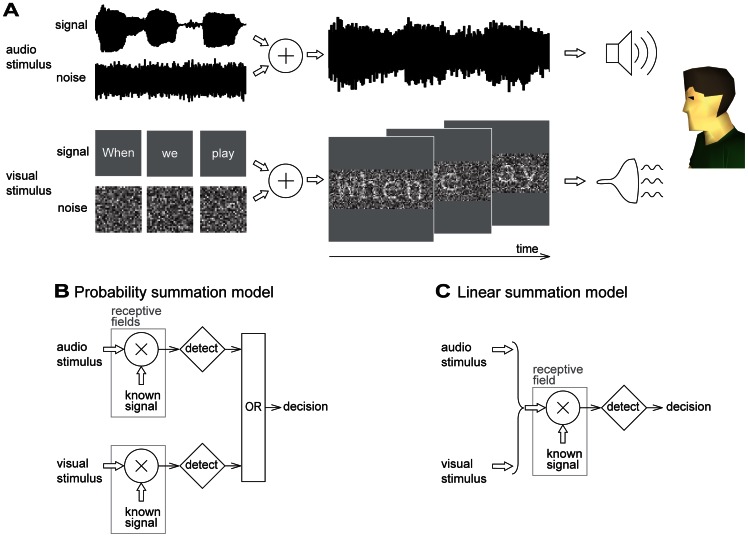
Materials and Methods. (**A**) A sentence (or a word) is presented as two concurrent streams: text and speech in visual and audio white noise. The observer identifies the words. In Experiments 1, 3, and 4, the visual stream includes only one word. In Experiment 2, the visual stream is a rapid serial visual presentation [35] of a sentence, presented one word at a time. The audio stream presents the same words as the visual stream. (**B & C**) The critical difference between models B and C is whether the two streams converge before or after detecting the signal. This dichotomy has been called “pre- and post-labelling” in speech recognition [36]. A neural receptive field computes a weighted average of the stimulus, i.e. the cross correlation of the stimulus and the receptive field’s weighting function [37]. In fact, if the noise is white, taking the weighting function to be a known signal, the receptive field is computing the log likelihood ratio of the presence of that signal in the stimulus, relative to zero signal. When the possible signals are equally probable, the best performance is attained by the maximum likelihood choice. (**B**) In probability summation, there is a receptive field for each possible signal. Detection occurs independently in each stream and the detections are combined logically to yield the overt decision. This is practically optimal when there is uncertainty among the known signals [38]. (**C**) In linear summation there is just one receptive field. The signals are linearly combined by a single audio-visual receptive field, followed by a single detector, which emits the final decision [22]. This is optimal for a known audiovisual signal.

Firstly, in ‘probability summation’, the cues are processed independently and then combined logically, e.g. detecting an object by any of several independent cues to its presence (Fig. 1B). This *preserves success*: if either component would have been successfully detected or identified independently, then the combination is detected or identified. Probability summation provides weak summation [29]. The strength of summation depends on the steepness of the psychometric function, which differs between hearing and vision, so the summation index of probability summation for detection is *k = *0.57 in vision and *k = *0.80 in hearing. (See ‘Probability summation and psychometric steepness’ in Materials and Methods.).

Probability summation had its heyday between 1920–1990. In hearing [30], it was worked out in 1921 (though not published until 1950). In vision [29], it was worked out in the 1960’s and 70′s. Probability summation remains the best account in hearing and vision for sensitivity to combinations of features [31]. It stipulates that a target can be detected or identified in any of several independent ways, so the probability of overall failure is the product of the separate probabilities (Fig. 1B). In hearing, this underlies Fletcher’s widely used articulation index [30], [32]. In particular, consider detecting a visual pattern composed of two spatial frequencies, low (L) and high (H), or identifying a spoken syllable whose waveform is filtered into two sound-frequency bands, low (L) and high (H). The probability *p* of detecting or identifying the whole is predicted by the measured probabilities of detecting or identifying the parts, 1−*p* = (1–*p*_L_)(1–*p*_H_) [30], [33], [34]. (Readers interested in false alarms should see ‘correction for guessing’ in Materials and Methods.).

The main alternative to probability summation is linear summation, which *conserves energy*, yielding strong summation [22]. Signal detection theory shows that the optimal detector of a known signal in white noise bases its decision on a cross correlation of the stimulus with the known signal [21]. This linear-summation strategy corresponds precisely to the popular model of a neuronal receptive field [37], which does not care how many cues or synapses there are, and treats the lot as one signal (Fig. 1C). (Of course, you might be surprised to be supposing a receptive field that spans vision and hearing.) This detector is ideal in that, on average, it performs as well or better than any other algorithm processing such stimuli. Signal detection theory shows that, for a given noise level, performance of the ideal is limited solely by signal energy [21], [22]. The ideal detector for a signal known exactly in white noise has 100% summation efficiency and a summation index *k = *1. It performs a linear summation. It *conserves energy*, in the sense that the total threshold energy for detecting the combination of two components is fixed, *E*_1_+*E*_2_ = *c*, if the two signals are limited by independent noises at the same noise level.

### Hearing and Seeing a Word

This paper is about the cost of combining: why we need more energy to perceive objects with more features. We focus on word identification, considering combining at high and low levels, e.g. between eye and ear, and between parts of a word.

We begin with the high-level combining of eye and ear before turning to the low-level combining of letters and syllables within a sense. For an evolutionarily established process, such as audiovisual localization [8] or recognizing the correspondence between facial and vocal expressions [39], one might expect performance close to the ideal of linear summation. Seeing the speaker’s lips move helps us understand spoken words [40] and sentences [41]. In contrast, for a more artificial perceptual task, such as reading and hearing a story, eye and ear might not integrate so well, and might even interfere. For example, while watching a foreign movie, native-language subtitles impair comprehension of the foreign speech [42].

We investigate the integration of concurrent speech and text, which is often seen in opera, television, movies, and the internet, especially when captioned for the hard of hearing. Concurrent speech and text is important in learning to read [18]. Our observers perform an intelligibility task on words and sentences presented through ear and eye (Fig. 1A). In Experiment 1, each trial presents, for 350 ms, a single word randomly selected from a set of 10 possible words. In Experiment 2, a trial consists of a sentence (average length 11 words), randomly selected from a set of 120 possible sentences, presented one word at a time, at a fixed rate (one word per 350 ms, i.e. 2.86 word/s). Observers verbally report the perceived words, taking as long as they like.

In both experiments, the audio and visual signals are presented in audio and visual white noise (Fig. 1A). Characterizing listeners’ comprehension of speech in noise is a classic problem in auditory research [43], and speech-in-noise exams are used extensively in clinical investigations [44]. Try our audiovisual demo (Fig. S1).

**Figure 2:**
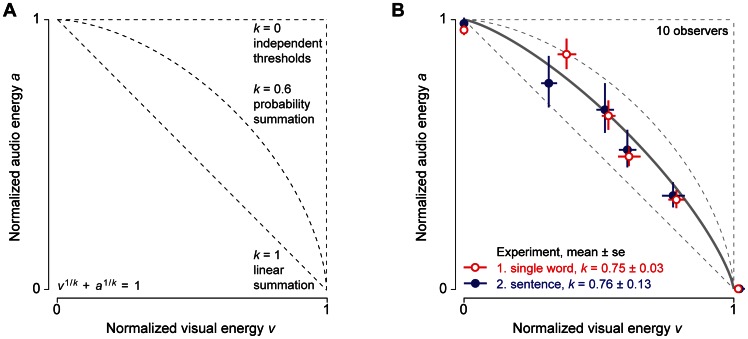
Predictions and Results. (**A**) Audio-visual summation is summarized by the summation index *k* of a smooth curve (Eq. 8) fitted to the threshold energies. The horizontal and vertical scales represent the normalized visual and audio energy components *v* = *V*/*V*_uni_ and *a* = *A*/*A*_uni_ of the bimodal signal at threshold. Each audio:visual ratio – including the two unimodal conditions (*V*_uni_, 0) and (0, *A*_uni_) – is a condition. All conditions are randomly interleaved, trial by trial (with one exception, described at the end of this caption). The noise is always present in both streams. For a given audio:visual ratio *A*/*V*, we measure the threshold (*V*, *A*) radially, along a line from the origin (0, 0). The curves represent degrees of summation ranging from none (*k* = 0) to complete (*k* = 1). The special case of *k* = 0 is to be understood as the limit as *k* approaches 0, which is max(*v*,*a*) = 1. (**B**) Averaging *k* over our ten observers, we find the same summation for reporting either a single word (*k = *0.75, red, Experiment 1) or a sentence (*k* = 0.76, blue, Experiment 2). The error bars indicate mean ± standard error. The curves obtained for each individual observer are shown in Figure S2. The virtue of randomly interleaving conditions (*a:v* ratios) is that the observer begins every trial in the same state, which enhances the comparability of the conditions plotted above. However, one might wonder how much better the observer would perform when the whole block is devoted to one condition. Random interleaving produces uncertainty; blocking each condition does not. Testing one observer (MD) on three conditions (audio, visual, and audiovisual signal; noise always present in both streams) we find insignificant difference in thresholds measured with and without uncertainty (i.e. interleaved vs. blocked conditions). Furthermore, ideal observer thresholds for the same conditions are negligibly different with and without uncertainty. This indicates that the results presented in this figure, found with uncertainty, also apply to performance without uncertainty.

The critical experimental manipulation is as follows: We characterize how well the observer combines by presenting stimuli with a total signal energy allocated in various proportions between the audio and visual modalities. We measure recognition threshold for six different audio:visual ratios. The extremes, with zero audio or zero visual signal energy, are *unimodal*. All allocation proportions, including the unimodal, are randomly interleaved (with one exception, described at the end of the caption to Fig. 2). From trial to trial, we adaptively adjust signal energy to estimate the energy required by the observer to correctly identify a word 50% of the time.

## Results and Discussion

Asking observers to integrate concurrent audio speech and visual text, three outcomes are particularly plausible. First, in *switching*, observers could attend to one stream and ignore the other (as some of our observers reported). Second, in *probability summation*, the observer might benefit from both channels merely because they provide two statistically independent ways to succeed (Fig. 1B). Third (and contrary to our observers’ impressions), in *linear summation,* the two streams could be treated as one combined stimulus (Fig. 1C).

To assess the cost of combining, we compare observer thresholds for the bimodal signals with those for unimodal signals. This paradigm allows us to distinguish between various proposals for how they are combined (e.g. Figs. 1B and 1C) by measuring the degree of summation, from none (*k = *0) to complete (*k = *1). Fig. 2A shows predicted curves for several values of *k*. Each threshold is plotted as a point in the two-dimensional space. The visual and audio components *v* and *a* of the plotted points are each normalized by the corresponding unimodal threshold energy [33].

We characterize summation by fitting a curve with an adjustable summation exponent 1/*k,*

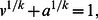
(8)where *k* is the *summation index*, *v* = *V*/*V*_uni_ and *a* = *A*/*A*_uni_ are the normalized visual and audio energy components of the threshold for a bimodal stimulus (*V*, *A*), and *V*_uni_ and *A*_uni_ are the unimodal visual and audio thresholds (*V*_uni_, 0) and (0, *A*_uni_). Some of the papers on summation talk about the just noticeable difference using just vision *σ*_V_, just hearing *σ*_H_, or both *σ*_VH_ (e.g. [45]). The optimal combiner conserves squared precision, 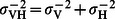. This is equivalent to our Eq. 8, with *k = *1. (*k* is 1, not 2, because *v* and *a* are energies, like *σ* ^2^.)

We fit Eq. 8 to the six threshold points in each experiment. We are interested in the degree of summation, especially in the range from none to complete (i.e. 0 to 1). Those two cases are illustrated in Fig. 2A. For *k = *1, energy sums linearly and the predicted thresholds lie along the negative diagonal. In the limit as *k* approaches zero, Eq. 8 becomes max(*v,a*) = 1, so the threshold for the bimodal stimulus is completely determined by whichever cue the observer is more sensitive to. Probability summation corresponds to intermediate values of *k*, around 0.6.

Zero (*k* = 0) and weak (*k* ≈ 0.6) summation represent distinct notions of ‘independence’. Zero *k* represents the case of independence of the two component values of the threshold. The stimulus is above threshold if and only if at least one of its components is above the corresponding unimodal threshold. This independence of threshold components corresponds to horizontal and vertical lines in our *a*-*v* plots. Probability summation (*k* ≈ 0.6) represents statistically independent success through either cue. Experiment 3 measures the unimodal audio and visual psychometric functions of three observers for the single-word task, calculates the bimodal thresholds predicted by probability summation, and fits Eq. 8 (see Materials and Methods). Probability summation predicts a *k* of 0.65, much less than 1, and is rejected by the human data.

Average results are shown in Fig. 2B. They demonstrate fairly strong summation of speech and text for single words (Exp. 1) and sentences (Exp. 2). On average, the summation index *k* is 0.76±0.13 for sentences and 0.75±0.03 for single words (mean ± s.e., ten observers). These values are significantly better than probability summation. The data from each observer are shown in Fig. S2.

Summation efficiency is the ratio of bimodal and unimodal thresholds. We find this ratio to be the same for single words and words in sentences. Sentence context typically improves performance in intelligibility tasks [46], [47]. Here, the introduction of sentence context is accompanied by an increase in the number of possible words from 10 to 404, which makes it harder to identify the word, and the net effect is to increase threshold (increasing log energy by 0.33±0.02, across all audio-visual ratios). However, as noted above, this change affects unimodal and bimodal thresholds equally, so their ratio, the summation efficiency, is unaffected (i.e. the effect of the different audio-visual ratios on the threshold difference between sentences and word presentation fails to reach significance: ANOVA with the audio-visual ratio as within-subject factor: *F*(5, 45) = 1.17, *p* = 0.34).

The efficient high-level combination of streams found here stands in sharp contrast to the inefficient low-level combination of features in recognizing words in either text or speech. As noted above, signal detection theory shows that, for a given level of white noise, the detectability of a known signal is wholly determined by its energy *E*. This ideal is plotted as the negative diagonal in Fig. 2: constant total energy, no matter how it is distributed between the two cues (with the proviso that we normalized each modality by its own threshold).

**Figure 3:**
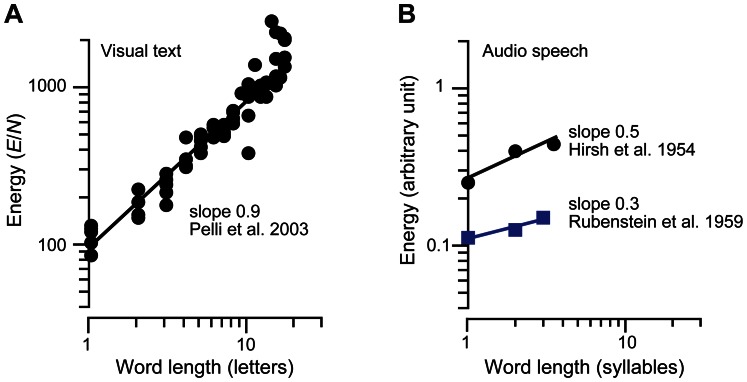
Assessing efficiency for combining the parts of a word: energy threshold as a function of word length. The summation index *k* is 1 minus the slope. Ideal thresholds, not shown, are independent of word length, with slope zero. (**A**) For a written word [24], the summation index is *k = *0.1. (**B**) For a spoken word [48], [49], the summation index is *k* = 0.5 or 0.7. See Methods for details.

In identifying a word, each letter or syllable is a cue, and one can measure the threshold energy for the word as a function of word length. Perfect combination predicts a fixed total energy, independent of word length (this would be a horizontal line in Fig. 3), but it is found, instead, that the threshold energy rises with a log-log slope of 0.9 (printed letters, Fig. 3A) or about 0.4 (spoken syllables, Fig. 3B), which corresponds to a summation index of *k = *0.1 (printed) or *k = *0.6 (spoken). This is inefficient combining, much worse than the ideal log-log slope of zero [24], [48], [49] and summation index *k* = 1.

### Efficiencies of Eye and Ear

Our theory, expressed in Eqs. 6 and 7, is about the overall efficiency to identify an object, *η*. Hearing and vision scientists have asked how well people recognize a spoken or printed word in noise. However, the audio and visual results were expressed differently, and have never been compared. (Comparing audio detection with visual contrast discrimination is apples and oranges, inconclusive [50].) Both fields measured the threshold (e.g. 50% correct) strength for identification of a word in white noise. Since Fletcher [32], hearing scientists have reported the signal-to-noise ratio in dB; this is (ten times the log of) the ratio of threshold signal power to noise power. Vision scientists, coming after signal detection theory, report threshold *E/N*, the ratio of signal energy to noise power spectral density.

The efficiency for detection of an optimized short sinusoid in white noise is about 20%, both in hearing [27] and in vision [28] (see ‘The efficiency for detecting a short sinusoid’ in Materials and Methods). Experiment 4 measures the efficiency of identifying one word, embedded in noise, presented either visually or auditorily. Efficiency was maximized by optimizing the voice and font of a four-letter one-syllable word. Remember that efficiency is the ratio of ideal and human threshold energies. Paralleling the similarity between eye and ear for detecting a short sinusoid, we find a low, roughly 1%, efficiency, at best, for identification of a spoken or printed word. This is for ten 4-letter one-syllable words, using the most-efficient voice and proportionally-spaced font (see ‘Experiment 4: Comparing the efficiency of eye and ear’ in Materials and Methods).

Thus, whether detecting a sinusoid or identifying a word, eye and ear are equally efficient. Efficiency for detecting the auditory or visual sinusoid is 20%, nearly as high as possible, and efficiency for identifying the word is roughly 1%.

### Summation Efficiency

Our low overall efficiency limits our lives. Identifying short words at roughly 1% efficiency, we need 20 dB more sound and ten times more visual contrast than the optimal algorithm. Eq. 6 allows us to express overall efficiency as a product, 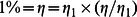. Note that the first factor, the one-feature identification efficiency, is greater than 20%, so the second factor, the summation efficiency, must be less than 5%. Thus, most of the cost in this word identification lies in combining the many features of the word.

**Figure 4:**
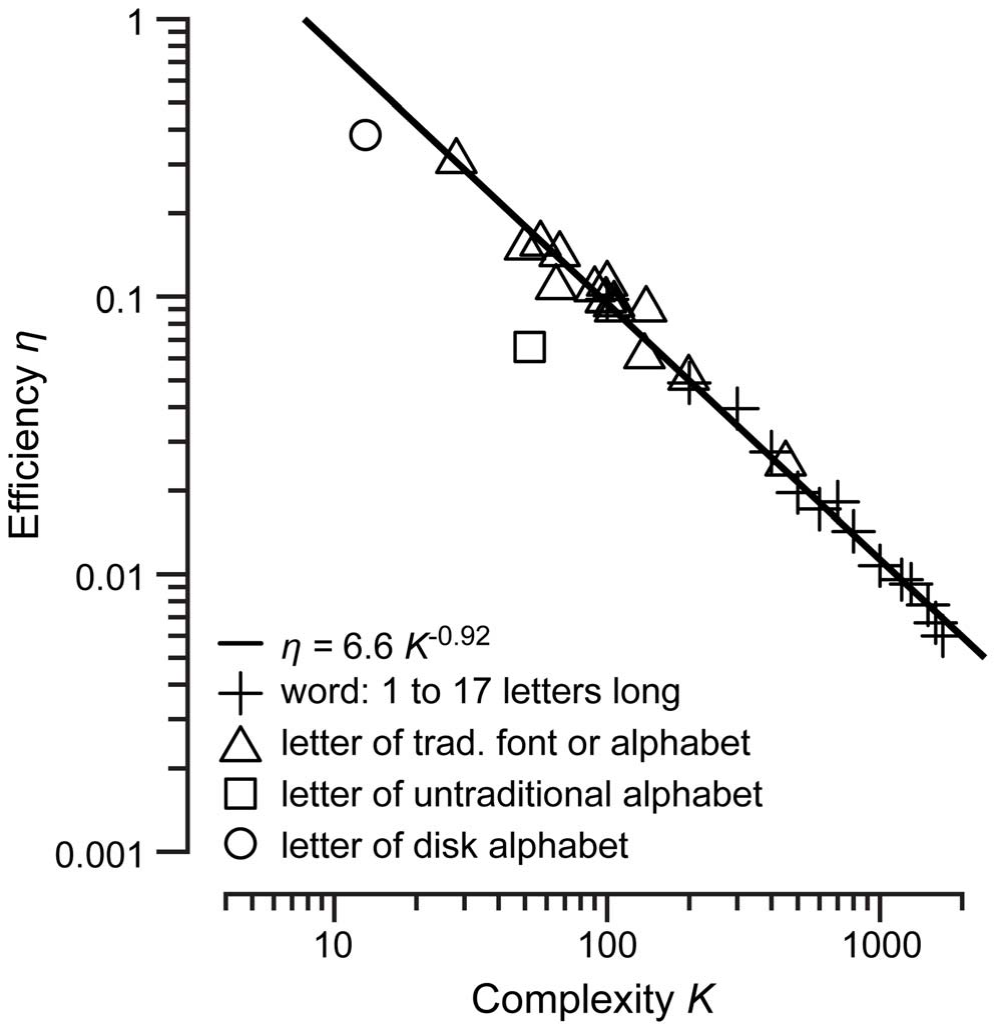
Efficiency for identifying letters and words as a function of their complexity. Efficiency is nearly inversely proportional to complexity over a nearly hundred-fold range. The horizontal scale is the perimetric complexity (perimeter squared over ink area) of the letter or word. Each**+**is efficiency for identifying one of 26 words of a given length (1 to 17) in Courier [24]. Each Courier letter has a complexity of 100 (averaging a-z), and the complexity of a word is proportional to its length. Each △ is efficiency for identifying one letter of one of 14 traditional fonts and alphabets by native or highly trained readers, in order of increasing complexity [25]: *Braille, bold Helvetica, bold Bookman, Sloan, Helvetica, Hebrew, Devanagari, Courier, Armenian, Bookman, Arabic, uppercase Bookman, Chinese, Künstler*. The outlying □ is efficiency for a letter in an untraditional alphabet: *4×4 random checkerboards*, after extended training [25]. The outlying ○ is efficiency for identifying the location of a disk. (See ‘Experiment 5. Identifying disks’ at the end of Materials and Methods.) A disk has the lowest possible perimetric complexity *K* = 4*π* = 12.6. A linear regression of log efficiency vs. log complexity for the traditional letters (13 fonts and alphabets) and words (13 lengths), excluding the untraditional alphabet and disk, has a slope of −0.92 and *R* ^2^ = 0.99. The regression line and its equation are shown.

This object recognition theory (Eq. 7) may seem too simple, even ridiculously so. One might expect observers to benefit from template-matching recognition of word shape, which would outperform our model. However, word shape contributes very little to reading speed, and, presumably, word recognition [46]. We presented Eq. 7 as a theory for efficiency (i.e. sensitivity in noise) for word identification, because that is what we have data for, but we see no reason why it should not apply to all objects. Yet our theory does not care about signal shape at all, beyond the feature count *n*. What about letters? Letters vary greatly in shape, and human efficiency for letter identification varies greatly among fonts and alphabets. Can our theory explain that? Yes. In Fig. 3, we supposed that the number of features is proportional to the word length, but we don’t know how many features per letter. Perimetric complexity (perimeter squared divided by ink area) is a plausible estimate of the number of features in a letter, to within a constant of proportionality [25]. This makes it very interesting to plot efficiencies for a letter (of any of several fonts) and for words (of several lengths) as a function of complexity (Fig. 4). This has a log-log slope of −0.92 and *R^2^* = 0.995.

Supposing that the number of features in an object is proportional to its perimetric complexity, we write *n* = *K*/*K*_1_, where *K*_1_ is the (unknown) complexity of a feature. Then our prediction, Eq. 7, becomes 
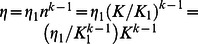
, which is identical to the regression line, 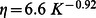, where the summation index *k* is 0.08 and the proportionality constant 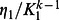 is 6.6. We have not been able to think of a way to factor 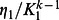, to separately measure the constants *η*_1_ and *K*_1_, but the data are well accounted for without knowing that extra fact.

### Strong vs. Weak Summation

**Figure 5:**
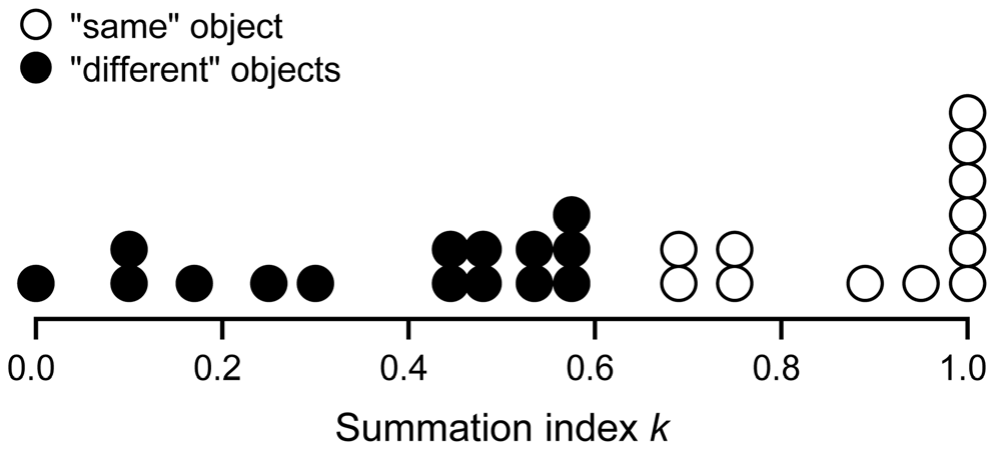
Histogram of the values of the summation index *k* reported in Table 1.

We report a dichotomy between the efficient high-level multi-sensory summation of text and speech and the inefficient low-level summation of word features within a sense. So, what make us summate efficiently or inefficiently? To achieve a broad perspective, Table 1 surveys the summation efficiencies from 23 papers: 7 cross-modal (including this one) and 15 uni-modal. Computed summation efficiencies are plotted in Figure 5. Table 1 and Figure 5 show that summation efficiency is high (near 1) if and only if the components are both perceived as the “same” object. This is brought out nicely in two papers that introduced cue differences that reduced the observer’s efficiency from high to low. Gepshtein et al. [51] introduced a spatial offset between their visual and haptic objects. When coincident, the two are perceived as one object and summate efficiently (*k* = 0.84, note that Table 1 only lists the average of Gepshtein and Banks [52] and Gepshtein et al. [51]). When sufficiently displaced, the two are perceived as distinct objects and summate inefficiently (*k* = 0.17). Nandy and Tjan [26] replicated the classic finding that a low- and a high-spatial-frequency grating summate inefficiently (*k* = 0) and went on to discover that a low-frequency band of a letter summates efficiently with a high-frequency band of the same letter (*k* = 1). In both studies, summation is efficient (*k* >0.6) if and only if the two components are both perceived as different aspects of the same object, regardless of whether they are mediated by different senses.

Note that high summation efficiency is much more common in the cross-sensory summation studies (2 senses, upper half of Table 1) than in studies of summation within a sense modality (1 sense, lower half of Table 1). This counterintuitive result shows the brain summating more efficiently across senses than within a sense. However, that is a misleading consequence of which stimuli have been most investigated, an artifact of stimulus selection. The logic of summation studies usually supposes that the two components are limited by independent noises. For that to be plausible, the stimuli must be substantially different. To achieve that, studies of summation within a sense have usually used stimuli that represent different objects, e.g. adjacent gratings or letters in a word. Stimuli mediated by different senses are already different, freeing investigators to pick stimuli that appear to be the same object. However, the difficulty can be overcome. Table 1 includes several studies that found high summation efficiency for two visual cues (texture perspective and stereo disparity) to the same object.

Table 1, taken as a whole, dispels any past impression that summation efficiency is consistently associated with high-level or evolutionarily old tasks. Instead, what matters is whether the two components are perceived as the same thing (object). Rosch noted that what we perceive as an object is not arbitrary [66]. Thus the high- and low-frequency bands of an “a” each look like an “a”, but in a pair of high- and low-frequency gratings, neither alone looks like the composite plaid. When perceiving a word, speech and print combine efficiently because they are perceived as the same thing, the word. But adjacent letters or syllables combine inefficiently, because each is perceived as a distinct thing, not the word itself. It seems that combining likelihoods, for efficient summation, requires the infrastructure that both stimuli are perceived as the same object, activating the same mental category.

The conclusion that we draw from the Table is new: combining is efficient if and only if the two cues each independently code for (or appear to be aspects of) the same thing. The reports of both high and low efficiencies by Gepshtein et al. (across senses) and Nandy and Tjan (within a sense) are key to interpreting the table. However, they confined the scope of their reviews and presented much narrower conclusions. Nandy and Tjan emphasized the remarkable finding of efficient summation across frequency, not the importance of having cues that appear to be the same object. In reporting their finding, Gepshtein et al. said, “Presumably, signals should not be combined when they come from different objects.” That sounds like happy news only because they were focused on cross-sensory combination and were not thinking about the persistent finding of inefficient summation for neighboring objects within a sense, which their finding explains nicely. In fact, in recognizing a word, letters summate inefficiently, as though each were a distinct object. This is the sad side of the Gepshtein et al. finding.

One might suppose that the inefficiency of combining is inevitable. After all, how can we expect a self-wired brain to perform as well as a statistician who knows how to compute and combine likelihoods? And yet, the brain does achieve this optimal summation when the cues are perceived as the same thing, even if they arrive through different senses. When we combine efficiently, the brain is smart about the weights, adjusting them in the face of changing reliability to achieve optimal combination [19]. This statistical proficiency does not extend to combining cues that are perceived as different things. Thus, people can combine likelihoods optimally, but only for stimulus components that independently refer to the same object. This seems less like an all-powerful statistician and more like a specific combining glue, not applicable generally, that joins only the data that arrive to that particular object memory in the brain.

We have seen that identifying an audiovisual word involves inefficient combining of letters or syllables and efficient combining of senses. How does it work? Might the combining of senses bypass the inefficient combining of adjacent parts? That seems unlikely, since reading speed (i.e. serial word recognition) is mostly due to letter-by-letter decoding and much less due to recognition of word shape [46]. Thus the efficient combining of audio and visual word cues must build upon the inefficient combining of word parts.

Hearing and seeing the speaking of a word refer to the same speech act, whereas, in hearing and reading a word, the correspondence is abstract: They are the same word, but not manifestations of one human action. Intuitively, because hearing and reading seem so different, some of the authors expected that observers would switch streams, attending one or the other, and not integrate at all (as some observers later reported). However, our results show that observers do integrate the audio and visual streams, contrary to their own impressions.

And yet, the brain is not a student required to achieve the optimal solution by application of the proper method. Does it matter when its summation efficiency is low? That depends on the number of parts, or cues. An observer combining two cues with summation index *k* has a summation efficiency of 2*^k^* ^−1^, which is 78% when the index is low (*k = *0.65) and 100% when it is high (*k = *1). This is only a −0.1 difference in log threshold energy (−1 dB), which is piddling, too small to be of practical significance. However, most objects consist of many features. For example, a one-syllable four-letter word with perhaps 7 features per letter [25] means 28 features, which is many. For these short words, summation efficiency is a meager 1%/*η*_1_<5%. In other words, threshold energy is at least 5%^−1^ = 20 times higher than it would be with perfect summation. Thus low summation efficiency is innocuous for simple objects, but greatly impairs our ability to see complex objects.

Here we have found a rule governing the efficiency with which observers combine parts. Suchow and Pelli [67] show that the process of letter identification can be separated into two steps, detecting the parts and combining them. The ideal observer’s efficiency drops when forced to take two steps, but the human observer’s efficiency is unimpaired by this constraint. Detecting the parts separately is inefficient, but people do it anyway. Detection of each part is independent, with an efficiency independent of the number of parts. Human efficiency of combining many parts drops in inverse proportion to the number of parts, as though the observer combines only a small number of parts.

We suppose that our dichotomy between efficient integration across views of the same thing and inefficient integration across things corresponds to the fast and slow dichotomy of combining process described by Holcombe [68].

We have been concerned, here, with how well observers combine in summation experiments, assembling parts to recognize a whole. However, combining is not always good. In real life, and in masking and crowding experiments, the observer must combine selectively to isolate the object from masks and neighboring clutter.

### The Unitary Hypothesis

Our notion of efficient combination only of cues that independently code for the same thing is very similar to the “unitary” hypothesis that helps to explain the ventriloquist effect. A ventriloquist sits on the stage with a dummy on his knee. When the ventriloquist speaks, restraining the motion of his lips, but making the dummy’s mouth move, we mistakenly perceive the speech as coming from the dummy’s mouth. This ventriloquist effect is the mislocalization of the source of a sound for which there are discrepant audio and visual cues. It still works with minimal stimuli: simultaneously presenting an auditory beep and a visual mark at various locations along a line. If the audio-visual discrepancy is small then the two stimuli are fused, perceived as one event, and observers combine information from the two senses optimally in estimating its position [8]. However, at larger separations, the two are seen as distinct events and there is no longer a benefit in localization of either [69]. In their wide-ranging review of cross-sensory integration of discrepant cues, Welch and Warren [70] suggested that the fusion is enabled by a perceptual assumption of unity, that both senses were reporting on the same event. Bayesian modeling that explicitly includes this unitary hypothesis (one vs. multiple events) gives an excellent account of several aspects of the ventriloquist effect over the full range of discrepancy, opting for unity at small discrepancies and multiplicity at large discrepancies [71], [72]. In that context, of merely judging the location of a simple event or two, the brain performs splendidly, properly considering the two possible situations (one vs. two events) and computing optimally in each. Alas, in word recognition, the brain seems less splendid, as it inefficiently combines adjacent letters to recognize the word. This virtue and vice both reflect the same operating rule of efficiently combining only cues that code for the same thing.

### Conclusion

Sensitivity has been a central topic of perception research since the nineteenth century, yet it remains largely unexplained. We show here that, in identifying a word, spoken or printed, most of the cost in sensitivity lies in combining its many features. Whether presented to eye or ear, efficiency for detecting a short sinusoid with few features is a substantial 20%, whereas efficiency for identifying a word with many features is a mere 1%. Efficiency is nearly inversely proportional to complexity over a nearly hundred-fold range.

Past studies have found low and high efficiencies of summation in various cue-combination tasks. Our survey includes many summation studies whose results are consistent with the idea that object thresholds are mostly determined by inefficient combining of features. However, in the interest of making the task simple, well specified, and objective, these studies mainly use the tasks of pattern detection or parameter estimation (e.g. slant or thickness). It is hard to generalize from those tasks to object recognition. Recognizing words is an important object-recognition task, essential to everyday life. The beauty of our text-and-speech task is that, while showing strong summation across senses at the word level, it also shows inefficient summation over the length of the word, so that both findings, strong and weak summation, are simultaneously present. Understanding a word, written and spoken, exhibits both at once: the observer combines parts (letters and syllables) inefficiently while combining senses efficiently.

Only components that are perceived as the same thing are combined efficiently. Observers give different names to adjacent letters in a word, and combine them inefficiently. Observers give the same name to a word’s image and sound, and combine them efficiently. Thus the brain has the statistical machinery to optimally combine cues, but only for cues that are independently perceived as the same thing. Presumably such cues find their own way through the brain to arrive at the same object memory.

## Materials and Methods

### Ethics Statement

This study has been approved by the New York University Institutional Review Board, and conducted according to the Declaration of Helsinki. All human participants gave written informed consent. For observers under 18, parental consent was also obtained.

### Experiments

Experiments 1 and 2 assess summation of text and speech, respectively for single words and sentences. Experiment 3 measures the unimodal psychometric functions for word identification, to allow us to calculate the summation index *k* predicted by probability summation. Experiment 4 compares efficiencies for identifying single spoken and written words. Experiment 5 measures the efficiency for identifying the least possible complex visual object: a disk.

### Observers

Twenty native English-speakers (17–59 years) participated in the study. Ten of the observers took part in Experiments 1 and 2, three in Experiment 3, five in Experiment 4, and two (MD and DGP) in Experiment 5. All reported normal hearing and normal or corrected-to-normal vision. One participant in Experiment 4 exhibited an abnormally high audio threshold, diverging by more than 4 standard deviations from the average of the other participants. She was referred to an audiologist and her data excluded from the analyses. MD and DGP are authors. Other observers were naive to the purpose of the experiment. All participants (but MD and DGP) were paid for participating.

### Apparatus

The observer binocularly views a gamma-corrected grayscale CRT monitor (ViewSonic UltraBrite E90f+) from a distance of 57 cm, using a chin rest. The background luminance is set to the middle of the monitor’s range, about 70 cd/m^2^. The display resolution is set to 1024×768 pixels, 35.2×26.4 cm, i.e. 29.1 pixel/deg at 100 Hz refresh rate. Display characteristics differ for Experiment 5, and are reported below (see ‘Experiment 5 (Fig. 4): Identifying disks’). Stimulus presentation is driven by MATLAB running on an Apple Macintosh computer using the Psychtoolbox [73], [74], [75]. The observer wears stereo headphones (Sony MDR-V500) and the same audio signal is presented to both ears.

### Intelligibility Tasks: Word and Sentence Identification

Our task, identifying synchronous text and speech, allows us to concurrently manipulate the signal-to-noise ratio in two senses.

The observer fixates the 2.5° gap between two vertically aligned white bars (each 1°×0.07°) presented on the grey background at the center of the screen, and initiates the trial. Then a randomly selected stimulus (signal in noise) is presented through eye and ear. The fixation bars remain on the screen during the presentation of the stimulus.

The stimulus consists of two synchronized streams, visual text and audio speech, each embedded in zero-mean white Gaussian noise. In the *sentence* experiment (Exp. 2), the visual stream is a sentence presented sequentially, one word at a time, at a fixed rate (350 ms/word, 2.86 Hz, see below), each centered at fixation. There is no temporal gap between successive visually presented words. In the *single-word* experiments (Exp. 1, 3, and 4), the visual stream includes just one word, presented for either 350 ms (Exp. 1 and 3) or 470 ms (Exp. 4). The audio stream presents the same words, recorded with natural stress and intonation. At the end of the trial, the observer reports the signal.

For sentences, the observer verbally reports the perceived words, taking as long as he likes. The total number of accurately reported words per sentence is recorded, irrespective of word report order. Due to large variations in their audio durations, the last word of each sentence is not scored. The single-word experiments use a 10-alternative forced choice procedure. Every trial uses the same list of 10 possible words. A response screen appearing 100 ms after stimulus offset contains the 10 possible words, displayed at the same size, font, and contrast polarity as the word in the stimulus. The observer indicates which word he thinks was presented by using the mouse to move the cursor and click on the word.

### Stimuli

In Experiment 2, 120 sentences composed of 9 to 15 one-syllable words (11.3±1.4, mean ± s.d.) are used (word length 4.1±1.1 characters). Twelve of the sentences are from Kwon et al. [76], we composed the rest for this study. The sentences are easy to comprehend, roughly corresponding to first-to-fourth-grade difficulty level (mean Flesch-Kincaid Index 0.61, mean SMOG index 3.13, mean Gunning-Fog Index 4.51). Here are two of the sentences that we use:

“The two friends did not know what time the play would start”.

“On top of the pile there were two small pens”.

Experiments 1 and 3 use the ten most frequent one-syllable 3-letter words, according to Kučera and Francis [77]: and, but, can, had, has, him, his, not, one, was. Experiment 4 uses ten high frequency one-syllable 4-letter words selected from the Northwestern Auditory Test No. 6 (NU-6), Form A [78]: bath, deep, late, life, mess, ring, road, soap, talk, turn.

Written stimuli are rendered as light letters on the gray background. Experiments 1, 2 and 3 present words in lowercase Courier, a monospaced serif font, at normal spacing. Experiment 4 uses lowercase Helvetica, a proportional sans-serif font. The first letter of each sentence is capitalized and the period at the end is removed. None of the sentences contain punctuation marks. We use a fixed 1° visual angle x-height, the vertical extent of the characters with no ascenders or descenders. The signal is a word-shaped luminance increment. Its Weber contrast *c* is the ratio of the increment to the background luminance.

A linguistically trained female native Canadian speaker of English read the sentences (sampling rate 22,050 Hz, 16-bit resolution). To facilitate the synchronization of the visual and audio signals, the speaker is paced with a visual metronome (flashing dot). The speaker is instructed to read the sentence with natural stress and intonation, while producing words at the metronome rate (constant rate of 2.86 Hz, appropriate to elicit naturalistic speech).

Soundtrack editing was performed using PRAAT [79]. We align the two streams by first synchronizing the audio-visual onset of the first word and then stretching or compressing the audio stream (using the Time-Domain Pitch-Synchronous Overlap-and-Add algorithm [80]) to achieve alignment of the onset of the last word. The compression is 0.99±0.05 (mean ± s.d.). The overall word onset asynchrony (6±93 ms, mean ± s.d.) is well within the temporal audio-visual integration window [81].

The same speaker recorded single words used in Experiments 1 and 3 (in sentence-like context, minimizing co-articulation). The sound files used in Experiment 4 are edited from a recording of the NU-6 words, commercially released by Auditec, St Louis [82]. Word audio duration varies (Experiments 1 and 3: mean: 333 ms, range: 275–359 ms; Experiment 4, mean: 401 ms, range: 344–462 ms). The intensity of each sound file (sentence and single word) is peak normalized at 99% of its maximal amplitude and scaled to 70 dB SPL.

### Noise

Noise is added independently to each pixel of the visual stimulus. Each noise sample is a luminance increment or decrement from a zero-mean Gaussian distribution truncated at ±2.5 standard deviations (SD is 0.4 contrast). The root mean square (rms) contrast of the noise is 0.38. At the viewing distance of 57 cm, each noise check side (1 pixel) subtends 0.0344°. Visual noise is static, independent for each word. Zero-mean Gaussian white noise is added to each speech signal sample (sampling rate 22,050 Hz). Average (rms) audio noise sound pressure level is 70 dB.

### Thresholds

Threshold energies are estimated by the improved QUEST Bayesian adaptive procedure [83] with guessing rate γ = 0.002 for the sentence experiment, 0.1 for the single-word experiments, and lapsing rate *δ = *0.01. The steepness parameter *β*, which affects the speed of convergence but not the mean threshold estimate, is 3.5 for Experiments 1 and 2. In Experiment 3, we measured *β* to be about 1.5 and used this value in Experiment 4. To minimize standard error, all reported thresholds are based on a reanalysis of the trial-by-trial responses with *β = *1.5.

We record the power threshold estimate (mean of the posterior probability distribution) corresponding to 50% correct word recognition. Threshold energy is the average word energy (across all the words) at the threshold power. Each individual threshold estimate is based on about the same number of words: 5 sentences (each made of about 11 words, on average) or 55 single words.

Each experiment has several blocks of trials (about 330 words per block). In each block, several thresholds, one per condition, are simultaneously estimated using interleaved QUEST procedures. Thus, the observer never knows whether the next trial will contain visual or audio signals, or both. (Our one exception to interleaving is described at the end of the caption to Fig. 2.) At least four threshold estimates are obtained for each condition. The first threshold is taken to be practice and discarded. We compute the geometric mean of the remaining threshold energy estimates.

### Experiments 1 and 2: Assessing Summation

We measure the threshold energy required by the observer to achieve 50% correct recognition, under unimodal and four bimodal conditions. Each condition, including the unimodal extremes, has a different audio:visual ratio of energies.

The curve
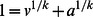
(M1)is fitted to the visual *v* = *V/V*_uni_ and audio *a* = *A/A*_uni_ components of the thresholds for the six conditions. The curve has three free parameters: the unimodal threshold energies *V*_uni_ and *A*_uni_ and the summation index *k*. Eq. M1 is fitted to the six threshold estimates, independently for each observer (*n* = 10) and experiment, by minimizing the squared error in log energy (between fit and data) along the radial line along which threshold was measured:



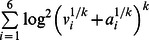
(M2)Supposing that the error in log-threshold measurement is fixed-variance Gaussian, minimizing this metric finds the maximum-likelihood fit.

### Probability Summation and Psychometric Steepness

The steepness of the psychometric function varies among tasks and senses. Vision papers usually present essentially the same analysis, but apply the exponent *β* to signal contrast amplitude instead of signal energy [29]. Energy is proportional to power, i.e. amplitude squared, so our summation exponent here is half theirs, 1/*k* = *β*/2, and the typical value of *β* = 3.5, generally attributed to probability summation, corresponds to a summation index of *k* = 2/3.5 = 0.57 here. Psychometric functions are sometimes expressed in terms of *d’*. In vision *d’* ∝ *c* ^2.8^ ∝ *E* ^1.4^ [84]. In hearing, *d’* is proportional to energy, *d’*∝ *E* [56], which corresponds to a summation index of *k = *0.8. Legge and Viemeister [85] make a closely related point, comparing vision and hearing.

### Experiment 3: Testing Predictions of the Probability Summation Model

We measured the psychometric function (i.e. performance vs. energy) of three participants for identifying unimodal (auditory or visual) single words. The signal energy of unimodally presented single words is varied according to the method of constant stimuli. For each modality, we use 15 energies uniformly spaced on a log scale (visual *E/N*: 25 to 2,500, audio *E/N*: 50 to 15,000, at least 30 trials per energy level). Visual and audio presentations are tested in separate blocks, and energies are randomized within each block. We fit a cumulative Gaussian psychometric function to the probability of unimodal word recognition as a function of the log *E/N*.

Let 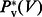 be the guessing-corrected probability (see below, ‘Correction for guessing’ and Eq. M4) that a word presented unimodally at a visual energy *V* is identified and let 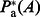 be the guessing-corrected probability that a word presented unimodally at an audio energy *A* is identified. The prediction of the probability summation model for the same word presented bimodally at energy (*V*, *A*) is
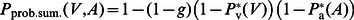
(M3)that is, one minus the probability that none of the three processes (two sensory channels and guessing) identifies the word.

For each participant, we compute the thresholds predicted by probability summation for bimodal presentations at various ratios of signal energy in the two modalities (Eq. M3). The summation index *k* is estimated by fitting Eq. M1 to the predicted threshold energies. Probability summation predicts an average *k* value of 0.65±0.03 (mean ± s.e., *n* = 3), which is much less than 1. For ten human observers presented with the same material, we find (Fig. 2B) that *k* is 0.75±0.03. Our results reject probability summation (one-tailed *t*-test, 7.7 d.f., *p = *0.029).

### Correction for Guessing

A cosmetic difference between the vision and hearing treatments of probability summation reflects the emphasis on detection in vision (two possible responses, with a nontrivial guessing rate) and on identification in hearing (many possible responses, with a negligible guessing rate). Nonzero guessing rate (i.e. occasionally correct answers despite zero signal energy) is accounted for by supposing a random independent deaf-and-blind guessing process with proportion correct *g*. Correction for guessing yields corrected probabilities
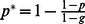
(M4)that are well described by the same summation formula
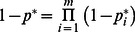
(M5)in both hearing and vision, where m is the number of independently processed components.

### Efficiency of Detecting a Short Sinusoid

In hearing and in vision, we do not have a complete description of the elementary features that mediate word identification. A short sinusoid is a plausible candidate as one feature, since it is fairly well matched to the tuning of auditory and visual receptive fields measured physiologically and tuning functions measured psychophysically. If observers detect a simple signal by template matching (a.k.a. cross correlation or feature detection) then the internal template can be discovered by adjusting the signal to maximize the observer’s efficiency [86]. Doing this for detection of a short sinusoid in white noise, it is found that the efficiency for detecting this candidate feature is about 20%, both in hearing [27] and in vision [28]. That is nearly as high as possible, given the steepness of the human psychometric function. As explained below, the steepness of the human psychometric function precludes efficiency higher than 24% (vision) or 33% (hearing) at the usual threshold criterion, *d′ = *1 (see below, ‘Efficiency of detection is limited by psychometric steepness’).

Please do not be distracted by the several higher efficiencies that appear in the literature. They are either not for detection of a known signal or wrong. Barlow [87] found high efficiency for detecting mirror symmetry, but that is a fundamentally different kind of task, a second-order task, requiring comparison of the image to itself, whereas detection of a known signal requires comparison of the image to a template (receptive field). Similarly, Kersten [88] reported high efficiencies for detecting visual noise, but this too is second-order, and cannot be performed by a linear receptive field. High efficiencies have been reported for contrast discrimination [50], [89], but that is not detection. The threshold contrast for contrast discrimination on a near-threshold pedestal is about one third of the threshold for detection [84], [90]. This makes efficiency for contrast discrimination about ten times that for detection, so the two efficiencies are apples and oranges, not comparable. Finally, Parish and Sperling’s [91] letter identification results are relevant, but they accidentally plotted the square root of efficiency instead of efficiency, so their widely quoted peak efficiency of “42%” for letter identification is actually 0.42^2^ = 18%, in agreement with later estimates [25].

### Efficiency of Detection is Limited by Psychometric Steepness

For detection, the well-known fact that the human psychometric function is much steeper than that of the ideal implies that human efficiency cannot exceed roughly 30% at the usual threshold criterion of *d′ = *1. This is because the psychometric function has a stereotyped shape, *d′* ∝ *E^b^*, with log-log slope *b*, where *b = *0.5 for the optimal (ideal) algorithm [20], *b* ≈ 1 for human hearing [22, Figs. 7-4 and 7-5], and *b* ≈ 1.4 for human vision [84]. A limited number of trials usually limits the range of measurement to 0< *d′* <3. Efficiency is the ratio of ideal and human thresholds *η* = *E*_ideal_ */E*, at a given threshold criterion, typically *d’ = *1. Because the human and ideal psychometric functions have different log-log slopes, the efficiency depends on the threshold criterion, as we see by solving for ideal and human thresholds, *E*_ideal_ ∝ (*d′*)^2^ and *E* ∝ (*d′*)^1/*b*^, and computing efficiency *η* = *E*_ideal_ */E* ∝ (*d′*)^2−1*/b*^. By definition of the ideal observer, a human observer’s efficiency cannot exceed 100% at any criterion. If efficiency is 100% at *d′ = *3, the highest level measured, then at *d′ = *1 it will be less, because the psychometric functions diverge. To be precise, the efficiency at *d′* = 1 will be *η = *1^2−1/*b*^/3^2−1/*b*^ *= *3^−2+1/*b*^, which is an efficiency of 33% for hearing (*b = *1) or 24% for vision (*b = *1.4). Since efficiency cannot exceed 100%, finding an efficiency higher than 33% (hearing) or 24% (vision) at *d′ = *1 would imply a psychometric function that is shallower than is usually found.

### Experiment 4: Comparing the Efficiency of Eye and Ear

We assess human performance on an absolute scale for the single word experiments (Exps. 1 and 4). We pit the human against the ideal observer, the algorithm that achieves the best possible expected performance given the noisy stimulus and its statistics. *Efficiency* is the ratio of the ideal’s threshold energy to the human’s [20], [92]. This strips away the intrinsic difficulty of the task, exposing a pure measure of human ability.

The task is to identify one of many possible signals, embedded in visual and audio Gaussian noise. All signals have equal prior probability. We implement the ideal in software. The ideal compares the noisy stimulus to each of the possible noise-free signals, computes the likelihood [25], and chooses the most likely signal. We use the same testing software to measure human and ideal thresholds, using the optimal algorithm to implement the ideal observer.

For Experiment 1, the stimulus was one of ten one-syllable three-letter words spoken by an unpracticed speaker and printed in the Courier font. Signal-to-noise ratio (SNR) in dB is ten times the log of the ratio of threshold signal power to noise power. The audio and visual SNRs are −8.2±0.5 dB and −8.0±0.2 dB (mean ± s.e. of ten observers). The human log *E/N* thresholds are 3.05±0.05 and 2.22±0.02 and the ideal log *E/N* thresholds are 0.37 and 0.74, so the efficiencies are 0.2% ±0.02% (audio) and 3.4% ±0.2% (visual). However, to meaningfully compare auditory and visual word-recognition thresholds, we must consider the effect of the stimulus parameters. Happily, human and ideal thresholds are similarly affected by the number of possible words, so efficiency (their ratio) is relatively independent of set size [25]. However, in our daily lives, we recognize words in a wide range of voices and fonts. Voice and font have different effects on the human and the ideal thresholds, so efficiency is affected. Trying to compare best with best in the efficiency contest between ear and eye, we select the very-well-spoken male voice of NU-6 and the widely-used Helvetica font. NU-6 is the Northwestern Auditory Test No. 6, Form A, available from Auditec of St. Louis. Helvetica has the highest efficiency for one-letter identification of the commonly used fonts tested by Pelli et al. [25]. For ten one-syllable four-letter words in the NU-6 male voice, we measure the audio threshold SNR to be −15.2±0.8 dB (for four observers), which is only slightly (1 dB) better than published values, after correcting for differences in number of syllables and set size. (Sumby & Pollack [40] report −15 dB for identifying one of 8 possible two-syllable words, from which we estimate −14 dB for one-syllable words. Hirsh et al. [48] report −8 dB for identifying one of 200 possible one-syllable words, from which we estimate −14 dB for 10 words, based on the effect of set size reported by Sumby & Pollack.) The audio log *E/N* is 2.43±0.08 and the ideal log *E/N* is 0.35, so efficiency is 0.9% ±0.2%. For Helvetica, visual SNR is −7.4±0.4 dB, log *E/N* is 2.35±0.04, and the ideal log *E/N* is 0.50, so efficiency is 1.4% ±0.1%. Comparing efficiencies for word identification, Helvetica, at 1.4%, beats the NU-6 voice, at 0.9%, but we call it a tie between eye and ear, both at roughly 1%, because fewer voices than fonts were tested.

Given that efficiencies range over many orders of magnitude, it is remarkable to find the competition between eye and ear yielding efficiencies as close as 0.9% and 1.4%. The scientific criterion for saying that two things have the same value is that the results of a reasonable set of measurements cannot reject the null hypothesis that they are the same. In the present case we are comparing the best audio efficiency with the best visual efficiency. We find large effects of font and voice on efficiency. We chose the most efficient of the several fonts and voices that we sampled, but it is very likely that there exists another voice or font with 50% higher efficiency. Thus our data, thorough as they are, cannot reject the null hypothesis of equality of the maxima.

### Energy vs. Word Length (Fig. 3)

Each experiment measured the observer’s threshold for identification of a word in white noise. The experiment was visual [24] or auditory [48], [49]. The threshold criterion was 64% (Pelli) or 50% (Hirsh and Rubenstein) correct. The Pelli and Rubenstein experiments tested each word length in its own block, with a known list of familiar words: Pelli used 26; Rubenstein used 12. In each block, Hirsh’s list included 25 nonsense syllables, 50 one-syllable words, 75 two-syllable words, and 25 “polysyllable” words. Rubenstein used word lengths of 1, 2, and 3 syllables. Hirsh reported thresholds for one-syllable, two-syllable, and “polysyllabic” words. The “polysyllable” words had three or more syllables and we suppose an average length of 3.5 syllables. We omitted Hirsh’s spondee words and nonsense syllables. The Hirsh and Rubenstein words had a frequency of about 100 per 4.5 million words in printed magazines. Pelli used the most frequent 26 words at each length. Lacking access to the recordings, to directly measure the sound energy of each word, we estimate the energy, except for an unknown proportionality constant. The threshold speech-to-noise ratio *r*, in dB, is converted to power and multiplied by the number *n* of syllables, to get the energy
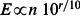
(M6)


### Experiment 5 (Fig. 4): Identifying Disks

In exploring the effect of complexity on efficiency for letter identification, one naturally wants to explore the full range of complexity. Assessed by perimetric complexity, the simplest object is a solid disk, a spot, with a perimetric complexity of 4π≈12.6, the lowest possible. We created an “alphabet” consisting of four “letters,” each of which is a disk. The disks differ only in position. Each disk has 1.1° diameter. The position of the center of each disk is (±0.3°, ±0.3°) relative to the center of the screen. Only one disk is shown at a time, but the average overlap of each disk with the other three possible disks is 58%, which is within the range of overlaps found for commonly used fonts [25]. On each trial, we present one “letter” in noise and asked the observer to identify it. We measure identification efficiency by a procedure that differs only slightly from that of Pelli et al. [25]. The static white noise covers a square region 1.7° by 1.7°, and the rest of the screen is uniform at the same mean luminance, except for a small black number label, 1 to 4, near each corner of the noise. The observer responds by typing the number of the corner in which he thinks the disk is. The static letter in noise is displayed indefinitely, until the observer responds by typing his choice: 1, 2, 3, or 4. Each correct response is rewarded by short beep. Each run is 60 trials. The contrast of the “letter” is adaptively controlled by Quest to estimate threshold, defined as 64% correct (*β = *1.4, *γ = *0.25, *δ = *0.01). At least 4 thresholds are estimated per observer, and averaged. Viewing distance is 50 cm. Luminance is 200 cd/m^2^. Table 2 presents the display characteristics used for each observer. To generate the display, we compute a small image array, containing a disk in noise, with independent noise in each pixel. This small image array is enlarged by pixel replication to produce the displayed image. Thus each array pixel is expanded to produce a uniform square check on the display. The noise is independent from check to check and from trial to trial. The noise distribution is Gaussian, truncated at ±2 standard deviations. The RMS noise contrast is 0.18. Across observers, the geometric-mean efficiency is 38%.

**Table 2:** Efficiency for identifying a disk “letter”.

Observer	Resolution (pix/deg)	Check size (pix)	Check size (deg)	Threshold (log contrast)	Threshold (log *E/N*)	Log efficiency
DGP	42	6×6	0.14×0.14	−1.11±0.03	0.99±0.05	−0.38±0.05
MD	35.5	4×4	0.11×0.11	−1.18±0.02	1.05±0.06	−0.45±0.06

Thresholds and efficiencies are reported as mean ± se.

## Supporting Information

Figure S1 **Text and speech demo.** Each movie-player box presents a sentence in noise. The first is just audio; the second is just visual; the third is audiovisual. It’s hard with audio or visual alone, and easier with both together. The demo works fine with speakers, but you’ll hear it better with headphones. Visual efficiency is higher for smaller letters, so you’ll see it better from farther.
(ZIP)

Figure S2 **Individual summation curves for each of the ten observers for Experiments 1 and 2.** Models’ predictions and averaged data appear in Fig. 2. The summation index *k* is the exponent of a smooth curve (Eq. 1) fitted to the normalized threshold energies. The curves represent degrees of summation ranging from none (*k* = 0) to complete (*k* = 1). Each error bar indicates the mean ± s.e. Note that GC’s *k* = 0.01 for sentences is an outlier, much less than the mean, across the ten observers, of 0.76±0.13; it may be relevant that GC is working in D.P.’s lab on stream segregation, and is thus trained to process streams independently.
(PDF)

Table S1 **Notes on our survey of summation.** For each paper, we explain how we estimated *k* for our Table 1.
(PDF)
