# Supplementary material for: Seeing and Hearing a Word: Combining Eye and Ear Is More Efficient than Combining the Parts of a Word
Source: PLoS One. 2013 May 29;8(5):e64803. doi: 10.1371/journal.pone.0064803 (PMC3667182; doi:10.1371/journal.pone.0064803)
Supplement: Figure S1 — Text and speech demo. Each movie-player box presents a sentence in noise. The first is just audio; the second is just visual; the third is audiovisual. It’s hard with audio or visual alone, and easier with both together. The demo works fine with speakers, but you’ll hear it better with headphones. Visual efficiency is higher for smaller letters, so you’ll see it better from farther. (ZIP) [file pone.0064803.s001.zip › figS1-preview.html]

Figure S1 preview


# Fig. S1 preview

|  |  |
| --- | --- |
|  | Sentence one |
|  |  |
| Audio.   Double click it. |  |
|  |
| Visual.   Double click it. |  |
|  |
| Audiovisual. Double click it. |  |
|  |

|  |  |
| --- | --- |
|  | Sentence two |
|  |  |
| Audio.   Double click it. |  |
|  |
| Visual.   Double click it. |  |
|  |
| Audiovisual. Double click it. |  |
|  |

**Figure S1. Text and speech demo.** Each movie-player box presents a sentence in noise. The first is just audio;
the second is just visual; the third is audiovisual. It's hard with audio
or visual alone, and easier with both together. The demo works fine with
speakers, but you'll hear it better with headphones. Visual efficiency
is higher for smaller letters, so you'll see it better from farther.
