# Supplementary figures and images for: Seeing and Hearing a Word: Combining Eye and Ear Is More Efficient than Combining the Parts of a Word
Source: PLoS One. 2013 May 29;8(5):e64803. doi: 10.1371/journal.pone.0064803 (PMC3667182; doi:10.1371/journal.pone.0064803)

Normalized audio energy  $a$

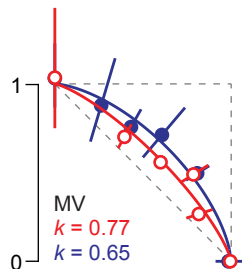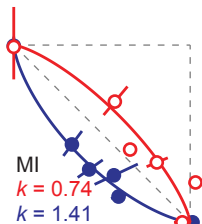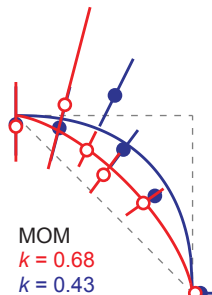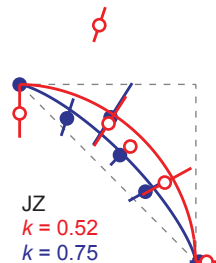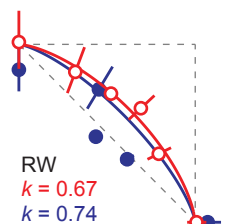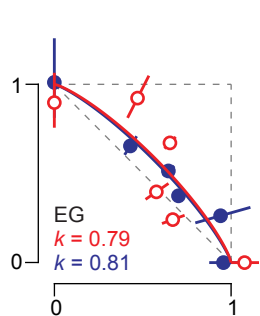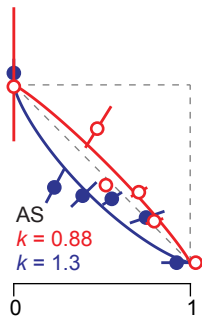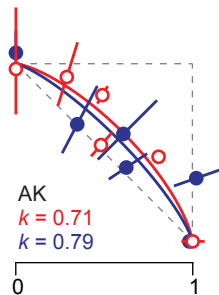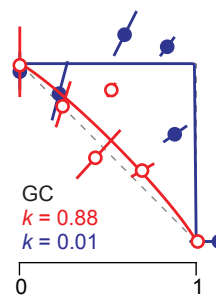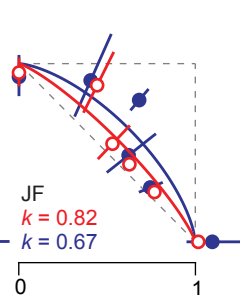

Normalized visual energy  $v$

Experiment —○— 1. single word —●— 2. sentence

Supplement: Figure S2 — Individual summation curves for each of the ten observers for Experiments 1 and 2. Models’ predictions and averaged data appear in Fig. 2. The summation index k is the exponent of a smooth curve (Eq. 1) fitted to the normalized threshold energies. The curves represent degrees of summation ranging from none (k = 0) to complete (k = 1). Each error bar indicates the mean ± s.e. Note that GC’s k = 0.01 for sentences is an outlier, much less than the mean, across the ten observers, of 0.76±0.13; it may be relevant that GC is working in D.P.’s lab on stream segregation, and is thus trained to process streams independently. (PDF) [file pone.0064803.s002.pdf]
