# Supplementary material for: Seeing and Hearing a Word: Combining Eye and Ear Is More Efficient than Combining the Parts of a Word
Source: PLoS One. 2013 May 29;8(5):e64803. doi: 10.1371/journal.pone.0064803 (PMC3667182; doi:10.1371/journal.pone.0064803)
Supplement: Table S1 — Notes on our survey of summation. For each paper, we explain how we estimated k for our Table 1. (PDF) [file pone.0064803.s003.pdf]

Publication	How we estimated k
**Arrighi et al. [53, Exp. 2]**	Their Bayesian fusion model (their Eq. 6) is misplotted in their Fig. 6. The axes are σ_A^{-2} vs. σ_V^{-2}, not σ_A^{-1} vs. σ_V^{-1}, so their Eq. 6 describes a straight line from (0,1) to (1,0). At the point where $\sigma_V = \sigma_A$, we compute the summation efficiency as 0.5 (σ_V^2 / σ_A^2) and use our Eq. 5, with n = 2, to compute k.
**Meyer et al. [54]**	We compute k from the values presented in their Figs. 6 and 8. We assume that their SNR thresholds are proportional to the variance of the noise. k = 1/b, where b is their summation index. This yields k values of 0.70 (the average of k estimates from their Experiments 1 and 2, 0.74 and 0.63, weighted by the number of participants) for aligned stimuli, and about 0.25 for non-aligned stimuli (non-aligned conditions in Exp. 1 and displacement larger than 30 degrees in Exp 2).
**Alais and Burr [8]**	We estimate k = 1 because they show that their results cannot reject optimal summation.
**Gori et al. [55, Fig. 4]**	We estimate k = 1 because they show that their results cannot reject optimal summation.
**Gepshtein et al. [51] ; Gepshtein and Banks [52]**	We extracted the averaged unimodal and bimodal JNDs, Δ_{unimodal} and Δ_{bimodal}, from Fig. 6 [51]. By design, the unimodal conditions have the same reliabilities (1/SD of the psychometric functions). For Gepshtein and Banks [52], we linearly interpolated (from their Fig 4b) the JNDs at the stimulus rotation at which visual and haptic stimuli have an equal JND and solved our Eq. 2 for k, $k = 2 \log_2(\Delta_{\text{unimodal}} / \Delta_{\text{bimodal}})$. For Gepshtein et al. [51], Δ_{unimodal} is the mean of the visual and haptic JNDs. The 'same object' k value in the table is the average of the estimates from the two papers (average k is 0.63 and 0.84, respectively, for Gepshtein and Banks [52], and the Gepshtein et al. [51] condition with 0 mm offset), weighted by the number of observers in each experiment. The 'different object' k is from [51], for conditions with a 90 mm offset (averaged over left and right displacements).
**Ernst and Banks [7]**	We estimate k = 1 because they show that their results cannot reject optimal summation: "In summary, we found that height judgments were remarkably similar to those predicted by the MLE integrator."
**Hirsh et al. [48]**	Their data are replotted in our Fig. 3.
**Rubenstein et al. [49]**	Their data are replotted in our Fig. 3.
**Green et al. [56]**	They report that the reduction of energy SNR obtained for the tones made of 16 frequencies is on average 0.4 dB more than expected for linear summation of d' squared (the paper asserts that $d' \propto E$). So the threshold reduction is $6.4\,\text{dB} = 10\log_{10}\left(\sqrt{16}\right) + 0.4\,\text{dB}$, and $k = -\log_{16}\left(10^{-6.4/10}\right)$ (our Eq. 2 solved for k).
**Pelli et al. [24]**	Their data are replotted in our Figs. 3 and 4.
**Pelli et al. [25]**	Their data are replotted in our Figs. 3 and 4.
**Näsänen et al. [57]**	Their Fig. 4, for two observers, shows log-log slopes of -0.49 and -0.54 relating efficiency to letter complexity. k = 1 + slope.
**Nandy and Tjan [26]**	We estimate k = 1 because their results cannot reject optimal summation.
**Nandy and Tjan [26, Appendix D]**	We estimate k = 0, corresponding to independence of thresholds, because their integration is consistent with threshold equal to the lower component threshold.
**Graham et al. [33]**	They report β = 3.5 on a contrast scale, so we estimate $k = 2/\beta$.
**Robson and Graham [29]**	They report β = 3.5 on a contrast scale, so we estimate $k = 2/\beta$.
**Watson [58]**	We computed the average β from the histogram in his Fig. 6, and computed $k = 2/\beta$. In Exps. 1 and 2, the average k is 0.39 and 0.43 for observer AM, and 0.47 and 0.5 for observer RP.
**Rovamo et al. [59]**	Their Fig. 4 shows a log-log slope of -0.7 relating efficiency to the number of cycles. We compute k = 1 + slope.
**Watson et al. [60]**	Averaged over all spatial frequencies, gains are 2.86 and 1.76 dB (their Fig. 3). There are two cues and those gains are sensitivity gains measured on a power scale (dB), so we solved our Eq. 2 for k, with n = 2, $k = -\log_2\left(10^{-\text{gain}/10}\right)$. They showed that at the threshold for detecting the presence of a very-slowly moving grating their observers could not identify the direction of motion, which strongly suggests that the oppositely moving components were both seen as a stationary grating.
**Knill and Saunders [61] ; Hillis et al. [62]**	We estimate k = 1 because their results do not reject optimal summation.
**Oruç et al. [63]**	We estimate k = 1 for the 6 (out of 8) observers whose integration did not differ from that of an ideal observer (with possibly correlated cues). We follow the authors in omitting the two observers who do not show any benefit of having multiple cues.
**Rivest and Cavanagh [64]**	In estimating k, we follow the authors in assuming equal weights for the single-cue location estimates.
